# Resistance to Growth of Molds for Wood Modified with Hydrophobic Hybrid Silica Gel Containing Copper Amine Complexes

**DOI:** 10.3390/ma14030577

**Published:** 2021-01-26

**Authors:** Shaokun Hao, Chuanshuang Hu, Xiuyi Lin, Jin Gu, Hong Yun, Weiwei Zhang

**Affiliations:** Key Laboratory for Biobased Materials and Energy of Ministry of Education, College of Materials and Energy, South China Agricultural University, Guangzhou 510642, China; shaokun@stu.scau.edu.cn (S.H.); lxysandy@outlook.com (X.L.); gujin@scau.edu.cn (J.G.); hongy@scau.edu.cn (H.Y.)

**Keywords:** poplar wood, copper complexation, resistance to growth of molds, hydrophobization

## Abstract

Complexation copper with amine provides an effective strategy for fixation copper in wood, while hydrophobic modification improves the dimensional stability of wood. Thus, a combination of complexation and hydrophobization is expected to enhance the efficiency of copper-based biocides. In this study, hydrophobic hybrid silica gel containing copper amine complexes (MACu) was prepared through an in situ sol-gel process in wood using methyltriethoxysilane (MTES), 3-amino-propyltriethoxysilane (APTES), and copper chloride. The resistance to growth of molds for MACu modified wood (*Populus tomentosa*) was measured according to ASTM D3273-16. A leaching resistance test was carried out in accordance with AWPA E11-16. The results showed that only *Aspergillus niger* covered the surface of untreated wood blocks and no mold grew on the MACu surface even after the leaching test. MACu xerogel and MACu wood were further characterized by SEM-EDS, FTIR, and XPS. A possible schematic diagram of the reaction mechanism was proposed to explain the high-efficiency anti-mold performance of MACu wood.

## 1. Introduction

The primary components of wood are cellulose, hemicellulose, and lignin, while the secondary components are complicated, including nutrients (such as starch and protein), kinds of extractives and natural oils, etc., which depends on the wood species. Some wood products are susceptible to biological degradation, i.e., mold fungi, decay fungi, and insects due to the difference of secondary components [1]. Therefore, wood protection against microorganisms is crucial for increasing the service time of wood products either used indoors or outdoors.

Copper is an effective constituent of fungicide for wood preservatives, such as chromated copper arsenate (CCA), alkaline copper quaternary (ACQ), copper azole (CA), etc., since it could denature the proteins and enzymes through its affinity for thiol groups in the fungal cell and inhibit the respiration by interference with the activity of conversion of pyruvate to acetyl coenzyme A [2]. The main disadvantage of these preservatives was the easy leaching of copper from treated wood, which greatly influenced their sustaining fungicide activity [3,4]. A combination of copper with chromium in CCA has satisfactory fixation to wood. However, CCA has been banned in Europe and North America due to its high toxicity to humans and the environment [5]. Another effective strategy for fixation copper ion is complexation where the copper fungicide activity was not significantly reduced. Palanti et al. modified wood with siloxane bearing amino groups with copper-linking function, and the results showed good resistance against brown rot and white rot fungi [5,6]. Copper fixation to the silica gel by complexation with the amine functions was further confirmed by solid ^29^Si NMR, electron spin resonance (ESR), and Scanning electron microscope-Energy dispersive spectrometer (SEM-EDS) investigations [7]. Bergamonti et al. prepared several polyamidoamines (PAAs), which showed a certain degree of protection against fungi and insects [8]. The PAAs could form a ligand/metal complex towards the Cu ion [9] and thus formulations of PAAs and copper applied on wood possessed improved biocidal activity even after a leaching procedure [10].

Mold attack is another important microbial invasion at wood that is easily ignored. Under high moisture and high temperature especially in tropical areas, mildew growth can occur on a variety of surfaces both of organic and inorganic origins, as fungal spores are present in air all the time [11]. Although mildew at wood does not decrease the structural integrity, it produces colored residue that can cause severe discoloration [11]. In extreme cases, mildew would threaten human health, causing skin allergy, and respiration tract infection [12]. Organic fungicides, such as 2-decyldimethyl ammonium chloride (DDAC), propiconazole, and tebuconazole 3-iodo-2-propyl-butyl carbamate (IPBC), etc. are very effective against most kinds of mold in a short time. However, poor chemical stability and durability inhibit their long-term anti-mildew application on wood [13]. In addition, organic fungicides are always toxic and harmful to humans and livestock. Recently, a number of studies have developed novel inorganic mildew preventives with lower toxicity, including nano-ZnO [14,15,16], TiO_2_ [17,18,19], CuO [20], graphene [21], Ag, or a combination of them [22,23,24,25]. These nano-sized inorganics form a protective coating layer at wooden products surface. The potential risk is that any physical damage or peeling of the coating layer would lead to mildew quickly since wood inside was not protected. Therefore, mold-resistant agents that can penetrate wood inside to protect wood in three-dimensional are more suitable for long-term anti-mildew.

Wood is a hygroscopic material with dry shrinkage and wet expansion characteristics so that cracking produces on the wood surface during weathering. As a result, it offers favorable conditions for spore germination and fungal decay [26]. Hydrophobic modification on wood would improve its dimensional stability and reduce cracking. Furthermore, microbial adhesion decreases with the increase of surface hydrophobicity [27]. In our previous work, it was proved that there was a synergistic effect between anti-mildew agent and hydrophobic surface that could enhance the anti-mildew efficiency [28]. A combination of complexation and hydrophobization is expected to enhance the efficiency of the copper–based biocides. Thus, in this study, a wood preservative containing copper amine complexes proposed by Palanti et al. [5,6] was improved to incorporate with a hydrophobic monomer methyltriethoxysilane (MTES). During a hybrid sol-gel process, copper ion was firstly complexed with 3-aminopropyltriethoxysilane (APTES) and then mixed with MTES to form a precursor solution. In the immersion process, hydrolysis and co–condensation took place in situ in wood lumen and cell wall. The anti-mildew test, copper leachability, microstructure, and surface chemistry of wood blocks were characterized and analyzed.

## 2. Materials and Methods

### 2.1. Materials

*Populus tomentosa* plank (Zibo, China, with air-dry density 450–500 kg/m^3^) was cut into blocks (L × T × R) of 30 mm × 30 mm × 5 mm. In addition, 3-aminopropyl triethoxysilane (APTES, 98%) and Methyltriethoxysilane (MTES, 98%) were provided by Aladdin Biochemical Technology in Shanghai, China. Copper chloride dihydrate (AR), acetic acid (AR), and ethanol were purchased from Guangzhou Chemical Reagent Factory and used without further purification.

### 2.2. Hybrid Sol-Gel Process for Wood Modification

Firstly, 1.16 mL APTES and 0.17 g copper chloride dihydrate were added into 100 mL ethanol solution under magnetic stirring for 30 min. The molar ratio of amino groups to copper ions was set at 4:1 for the desired complexation. Then, 11.24 mL MTES was added so that the weight ratio of APTES to MTES was 1:9. In addition, 2 mL acetic acid was further added to the above solution. Furthermore, 20 mL of the mixture solution was stored in a Petri dish covered with a lid for 3 days to form a pure MACu xerogel. Wood samples were pre-conditioned at 25 °C and 65% relative humidity (RH) for one week. The moisture content was measured to be 12% which was lower than the fiber saturation point so that it could avoid gel deposition in cell lumina [29]. Then, they were immersed in the above hybrid precursor solution at room temperature for 24 h. In order to increase the thickness of the coating layer at wood surface, another 5 times of the dip coating processes were carried out with the same solution, which included soaking for 5 min and then drying for 30 min at room temperature. Wood blocks modified with the hybrid solution were named as MACu wood. For comparison, the MTES solution without APTES and copper chloride was also prepared with the same procedure for wood modification, which was marked as M wood. After that, all modified wood blocks were dried in an oven at 65 °C for 12 h and then at 103 °C for 24 h. Finally, they are adjusted in an artificial climate chamber (MGC−400H, 20 °C, and 65%) until the equilibrium moisture content was reached.

### 2.3. Leaching Resistance Test

The leaching resistance of copper in MACu was tested according to AWPA E11-16 [30]. Six pieces of MACu wood blocks were placed in a beaker containing 300 mL deionized water and placed in a vacuum oven at −0.1 MPa for 20 min. The beaker was then sealed with plastic wrap and placed in an artificial climate chamber (28 ± 0.5 °C, 80 ± 2% relative humidity). The deionized water in the beaker was replaced at intervals of 2, 6, 24, and 48 h. Another 5 times of water changes were carried out every 48 h. After that, the test pieces were removed, dried, and then adjusted the water content as mentioned before anti-mildew test. The loss of copper could not be measured according to the mass difference before and after the leaching test because components dissolved out were much larger than that of copper in MACu. Instead, the change of copper content was measured through elements analysis using pure MACu xerogel treated (around 1 g) with the same procedure as MACu wood blocks. 

### 2.4. Resistance to Growth of Molds Test

Resistance to growth of molds was tested according to ASTM D3273–16 [31] with minor modification [28]. Mixed mold was cultured by exposing malt extract agar medium in air, and mold colony covered with medium after 4 days at room temperature. Next, the mycelium was carefully transferred into a spray bottle using 20 mL sterile water with 1–2 drops of Tween 80. Then, wood blocks were sprayed with the suspension and placed at 28 °C and 90% relative humidity for 4 weeks. Anti-mildew levels were divided according to the area of mold infection. Infection area of 0–25%, 25–50%, 50–75% and 75–100% represented 1, 2, 3, and 4, respectively, and 0 represented no defacement. Five groups of wood blocks (including unleached control wood, leached control wood, unleached MACu wood, leached MACu wood, M wood) were used to determine the anti-mildew level with 6 duplicate samples in each group.

### 2.5. Characterization

The wettability of original and modified wood blocks was characterized by water contact angle using Contact Angle System OCA20 (DataPhysics, Filderstadt, Germany). Graphs of 5 μL water droplets on three different areas were recorded at 10 s after the water droplet contacted with the wood block surface. Their WCAs were calculated by Young–Laplace fitting to obtain the average values. Six replicates were tested to obtain the average WCA values and their standard deviations. Scanning electron microscopy (SEM, Carl Zeiss Evo 18, Jena, Germany) with an energy-dispersive spectroscopy (EDS, Oxford, UK) were used to observe the distribution of element in wood block. X-ray photoelectron spectroscopy (XPS, EscaLab 250Xi, Waltham, MA, USA) was used to measure the fixation content of copper before and after the leachability test. Fourier Transform infrared spectroscopy (FTIR, PerkinElmer, Waltham, MA, USA) was used to analyze changes in some functional groups powder samples and Attenuated Total Reflectance Fourier transform infrared spectra (ATR-FTIR) (TE-SOR27, Bruker, Karlsruhe, Germany) on a wood sample surface.

## 3. Results and Discussion

### 3.1. Results

**Figure 1:**
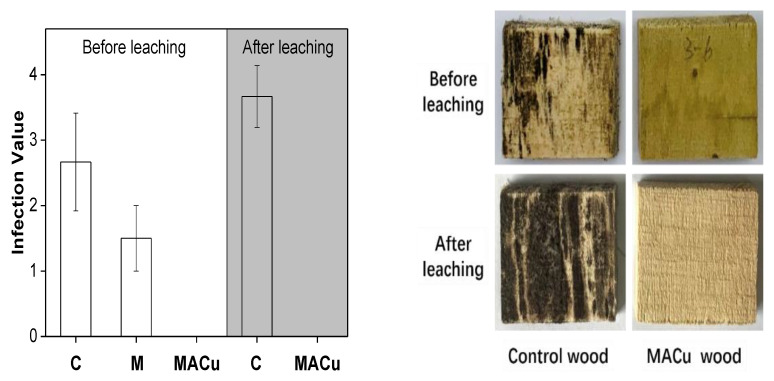
Resistance to growth of molds and typical appearance (after 4 weeks of mold incubation) for various poplar wood blocks before and after leaching tests.

After a 4-week incubation of mixed mold spores, the average infection value of the control wood blocks was 2.67, with the corresponding infected area 67%, and its typical appearance was shown in Figure 1.

When wood blocks were coated with M gel, the average infection value decreased to 1.5. No mold grew on the MACu wood surface. In order to detect the possibility of longterm anti-mildew performance, the leaching resistance of copper in MACu wood was tested. After 12 days of immersion in water, the control wood was almost completely covered with mold. In comparison, MACu wood revealed the original color of wood after the leaching procedure, and no sign of mildew was observed at its surface. Compared with the MACu wood before leaching, surface pale green (derived from copper ions) disappeared, indicating that some copper ions in MACu xerogel dissolved out. Nevertheless, resistance to growth of molds for MACu wood was not affected completely, demonstra-ting that MACu wood probably possessed excellent anti-mildew performance in an outdoor environment.

**Figure 2:**
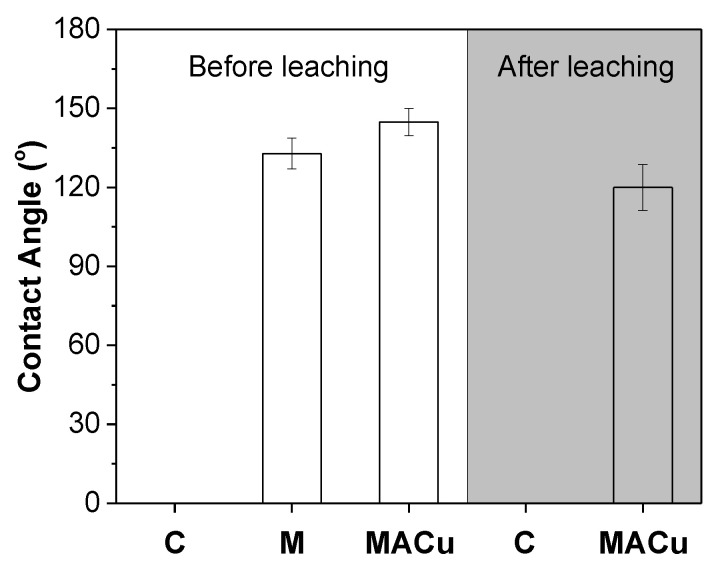
Hydrophobicity of various poplar wood blocks before and after leaching tests.

The hydrophobicity of various poplar wood blocks before and after the leaching test was measured, and the results are shown in Figure 2.

The poplar wood is highly hydrophilic since water droplets penetrated inside within 20 s. After M-gel modification, the contact angle of wood surface increased to 132.8 ± 5.8°. Coating with hybrid gel MACu, its contact angle further increased to 144.8 ± 5.2°. After the leaching resistance test, although the hydrophobicity of MACu wood declined, its surface was still regarded as hydrophobic, with a contact angle of 120.0 ± 8.7°.

**Figure 3:**
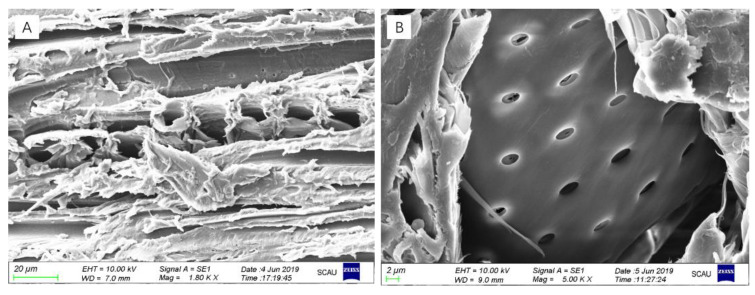
SEM images of MACu wood surface in (**A**) tangential section; (**B**) cross-section.

The surface morphologies of MACu wood was shown in Figure 3.

No obvious MACu xerogel layer or nanoparticles were observed on the wood surface. The coating layer was probably extremely thin as the main structure of fiber cell was clearly preserved, and the pore structure of wood ray was not blocked. The pit structure in the cell wall was still semi-open as seen in the cross-section in Figure 3B.

**Figure 4:**
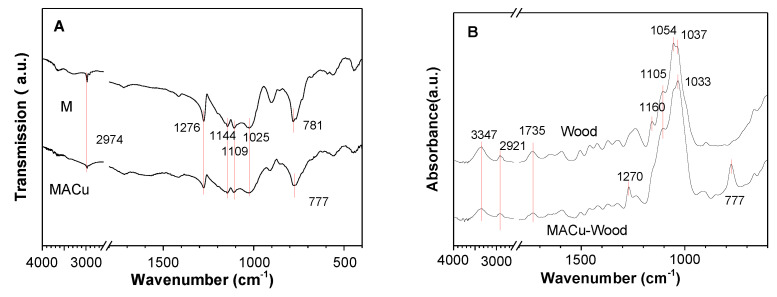
FTIR spectra of (**A**) M and MACu xerogel powder; (**B**) MACu-wood and wood.

The FTIR spectra of M and MACu xerogel can be seen in Figure 4A.

The peaks at 950–1150 cm^−1^, present on the spectrum of both M and MACu gel, contributed to Si–O–Si stretching vibration. The peaks at 2974 and 1276 cm^−1^, which belonged to stretching vibration and symmetric deformation of −CH_3_, weakened in MACu xerogel as compared to that in M gel. In addition, the peak at 781 cm^−1^ that belonged to the stretching vibration of Si–C [32] shifted to a lower wavenumber to 777 cm^−1^ in MACu gel. The ATR-FTIR spectra of wood blocks before and after silane treatments were shown in Figure 4B. A broad hydrogen bonded O–H stretching absorption at 3347 cm^−1^ and C–H stretching absorption at 2921 cm^−1^, 1735 cm^−1^ for unconjugated C=O in hemicellulose or carboxylic acid [33] were seen in infrared spectra of untreated wood. These wood characteristic peaks became less intense after MACu modification. On the contrary, peaks at 950–1150 cm^−1^ that belonged to C–O–C stretching vibration in cellulose and hemicellulose in the finger-print region of wood increased in intensity slightly, because they were overlapped with Si–O–Si stretching absorption as seen in Figure 4A. Furthermore, 777 cm^−1^ for Si–C stretching vibration in MACu gel was also observed after wood modification. The symmetric deformation of Si–CH_3_ in MACu-wood shifted to a lower wavenumber at 1270 cm^−1^, which might be explained as the overlapping effect with absorption peaks in the finger-print region of wood.

**Figure 5:**
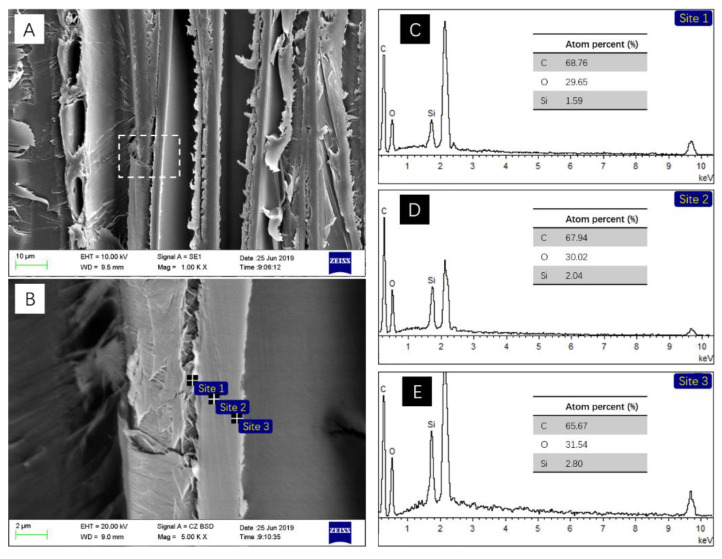
SEM images of MACu wood inside in tangential section (**A**) and elemental analysis of cell wall (**B**), where EDS (**C**–**E**) corresponded to sites 1, 2, 3, respectively, in (**B**).

Similar to the morphology of block surface, nano xerogel particles could not be observed in the cell lumens inside. SEM-EDS images (Figure 5B–E) of the cell wall in the radial section showed the presence of silicon, and its concentration decreased from cell lumen side to middle lamella. Copper and nitrogen elements were not detected probably due to their low contents in the precursor solution.

Copper and nitrogen elements were not detected probably due to their low contents in the precursor solution.

**Figure 6:**
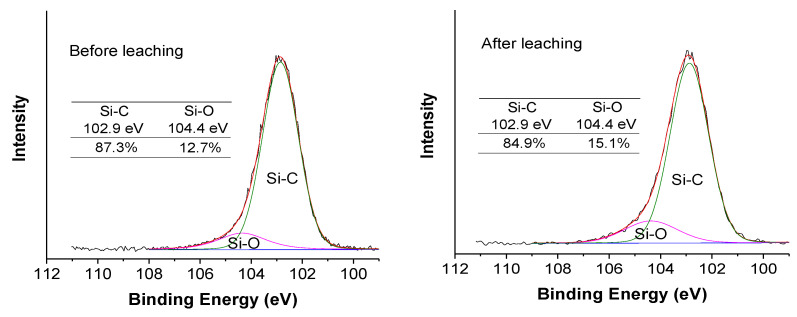
XPS survey of pure MACu before and after leaching.

XPS spectra of pure MACu before and after leaching resistant tests were performed to analyze the fixation of copper in the hydrophobic hybrid silica gel. Cu2p and N1s were detected in both samples, although their content was very low. The molar ratio of N to Cu increased from 2.46 to 3.99 in MACu powder after the leaching test. Another element change was also found with the ratio of Si/N also decreasing slightly, indicating that some silicon derived from MTES was hydrolyzed and dissolved out during the leaching test. In the high-resolution deconvolution of Si 2p peaks in Figure 6, peaks at 102.9 eV and 104.4 eV contributed to Si–C and Si–O, respectively.

The peak at 104.4 eV (Si–O) in the leached sample increased from 12.7% to 15.1% as compared with the pristine one.

### 3.2. Discussion

The population of mildew fungi depends on the fungal spores in air in different areas. In Guangzhou, which is located at the intersection of tropical and subtropical, the main molds include *Trichoderma viride*, *Aspergillus niger*, and *Penicillium citrinum,* and others. In previous anti-mildew study on bamboo and rubber wood [28], at least three kinds of mold were observed at their surface. Here, it seemed that only one mold, *Aspergillus niger*, grew on a poplar wood surface with a competitive advantage since nutrients in poplar wood are not as rich as rubberwood or bamboo.

According to the SEM images of MACu Wood in Figure 3, it was speculated that its surface was covered with a thin layer of MACu xerogel. It was confirmed by the FTIR spectra that wood characteristic peaks became less intense, while C–O–C stretching vibration in cellulose and hemicellulose in the finger-print region of wood increased in intensity slightly, because the latter was overlapped with Si–O–Si stretching absorption. CH_3_ groups in MACu xerogel decreased the surface energy of wood, thus increasing the contact angle. In addition, the original fiber structure at wood surface provided micron-sized roughness which further enhanced the surface hydrophobicity. The presence of silicon in the cell wall was confirmed by SEM-EDS in Figure 5. In this sol-gel process, no water was added to the precursor solution so that hydrolysis of alkoxysilane was suppressed in the precursor solution; thus, these unimoleculars were small enough to penetrate the cell wall [29]. The bound and free water in the wood then initiated the hydrolysis of the alkoxysilane to conduct the sol-gel process within the wood cell wall [34]. As a result, the diffusion process from cell lumen to cell wall resulted in the gradually decreasing distribution of silicon. Here, silicon in the cell wall was probably derived from MTES instead of APTES, as the latter formed an amino copper complex that increased the molecular size greatly. Hydrophobic modification both outside and inside of wood blocks had an inhibiting effect on the growth of fungus on wood, because the reduced rate and uptake of moisture in hydrophobic wood resulted in reduction or even prevention of diffusion of fungal metabolites into wood [35].

Excellent anti-mildew properties of MACu wood derived from not only the hydrophobic effect but also copper amine complexes in the hybrid gel. The molar ratio of N to Cu in hybrid xerogel increased to almost the same as the theoretical complex quantity of 4 after the leaching test, indicating that part of the copper ions was lost during leaching and the copper ions in amine complexes were stable against leaching. Part of the hybrid xerogel was found to be hydrolyzed during the leaching test since more Si–OH groups were produced derived from Si–O–Si [36]. Thus, the MACu wood block after the leaching test decreased its hydrophobicity due to the presence of these hydrophilic Si–OH groups. To be more precise, these Si–O–Si bonds were probably derived from MTES instead of APTES, so the ratio of Si/N also decreased slightly.

**Figure 7:**
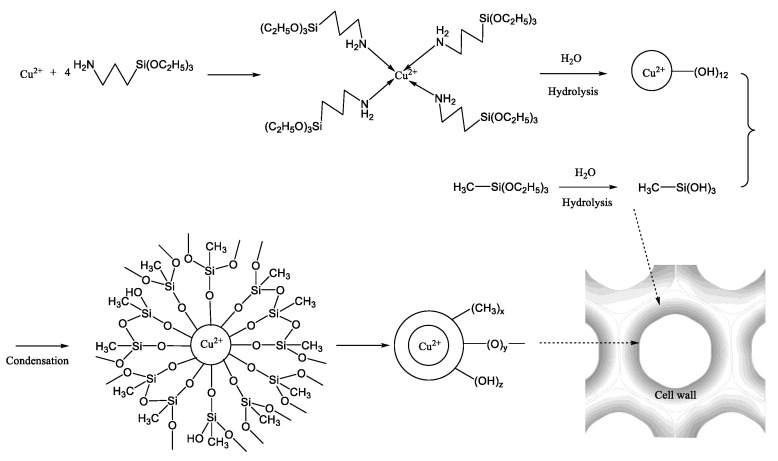
A possible schematic diagram of the reaction mechanism for MACu wood.

The sol-gel reaction of alkoxysilane has been reported in many research works. The hydrolysis rate of alkoxysilane was faster than its condensation rate in an acidic condition, thus sol formed in acidic solution had a better permeability. A possible schematic diagram of the reaction mechanism for MACu wood was proposed as follows: firstly, APTES complexed copper was formed in solution and then permeated into wood together with MTES. They were further hydrolyzed by water in a wood cell wall, respectively. A large amount of methyl silanol condensed with each other or at the surface of copper amine complex silanol (seen in Figure 7).

These oligomers formed a gel layer in cell lumen as well as the wood surface. Copper ions were well protected by hydrophobic methyl groups and Si–O–Si bonds so that copper ions in MACu showed excellent anti-leaching properties. In addition, part of the MTES molecules penetrated the cell wall and in situ sol-gel reaction took place. Methyl silanol became cross-linked with each other via Si–O–Si bonds and with hydroxyl groups in cell walls via Si–O–C bonds [37]. Even if spores on the wood surface began to produce hypha, the relatively “dry” and “toxic” environment in wood cell lumens would lead to the death of mycelium. Therefore, MACu wood showed durable hydrophobic and anti-mildew properties.

## 4. Conclusions

In this work, hybrid hydrophobic silica gel containing copper amine complexes was prepared through an in situ sol-gel process in poplar wood. A thin layer with nano-sized thickness of MACu xerogel formed to cover the wood surface. With the combination of CH_3_ groups in MACu xerogel and rough fiber structure, the wood surface showed high hydrophobicity with a water contact angle up to 144.8 ± 5.2°. The mixed silica gel was also found in the cell wall. MACu modified wood showed durable excellent resistance to growth of molds even after the 12-day leaching test because the copper amine complex was well protected by hydrophobic methyl groups and Si–O–Si bonds against leaching.

## Data Availability

Data sharing is not applicable to this article.
